# First European report of *Francisella tularensis* subsp. *holarctica* isolation from a domestic cat

**DOI:** 10.1186/s13567-020-00834-5

**Published:** 2020-08-31

**Authors:** Sonja Kittl, Thierry Francey, Isabelle Brodard, Francesco C. Origgi, Stéphanie Borel, Marie-Pierre Ryser-Degiorgis, Ariane Schweighauser, Joerg Jores

**Affiliations:** 1 Institute of Veterinary Bacteriology, Vetsuisse Faculty, University of Bern, Bern, Switzerland; 2 Department of Clinical Veterinary Medicine, Vetsuisse Faculty, University of Bern, Bern, Switzerland; 3 Centre for Fish and Wildlife Health, Vetsuisse Faculty, University of Bern, Bern, Switzerland

**Keywords:** bacteriuria, cat, feline, Switzerland, tularemia, zoonosis

## Abstract

*Francisella tularensis* subsp. *holarctica* is a select agent causing life-threatening tularemia. It has been isolated from humans and animals, mainly lagomorphs and rodents, rarely other wild carnivore species. Increasing numbers of human tularemia cases have been reported during the last 5 years in Switzerland. Here we report the first isolation of *Francisella tularensis* subsp. *holarctica* from a domestic cat in Europe and compare its genome sequence with other Swiss isolates. The cat isolate shows a close phylogenetic relationship with a contemporary hare isolate from close geographic proximity, indicating a possible epidemiological link.

## Introduction, methods and results

Tularemia is a zoonotic disease caused by *Francisella tularensis*, which comprises the four subspecies *tularensis, holarctica, novicida* and *mediasiatica*. The most virulent subspecies are *tularensis* and *holarctica*, which have been reported from North America and the whole Northern hemisphere, respectively [1]. The bacterium has a broad host range including different vertebrate groups as well as invertebrates [2]. Transmission occurs via inhalation of infected droplets, contact with infected animals, bites of arthropod vectors or oral uptake [3]. The isolation of the tularemia agent from European carnivores is restricted to isolated cases in free ranging wildlife. Reported cases include a stone marten in Switzerland (2012) [4], a raccoon dog (2012) and a red fox (2008) in Germany [5]. Regarding domestic carnivores, tularemia sporadically occurs in cats in North America where it is almost exclusively caused by subspecies *tularensis* [6]. In North America, disease occurrence in cats is considered an important factor for humans to contract tularemia [6]. Here we describe the unexpected isolation of *F. tularensis* subsp. *holarctica* from the urine of a domestic cat in Switzerland and compare the isolate to contemporary isolates from Swiss hares as well as previously published Swiss isolates [7] from wildlife, humans and ticks.

In March 2019, a 9 year-old male neutered outdoor cat was presented to the Small Animal Clinic of the University of Bern (SAC) for the routine 3-monthly maintenance flush of his bilateral subcutaneous ureteral bypass (SUB) placed 4 years ago. All cultures performed on previous rechecks had been negative. The owners reported no abnormalities and the clinical exam was unremarkable except for a reduced body condition score of 3/9 and a weight loss of 430 g over 3 months (actual body weight 3.47 kg). The cat had concomitant chronic kidney disease International Renal Interest Society (IRIS) stage 3 with a blood creatinine of 298 µmol/L (reference range: 52–138) and urea 26 mmol/L (reference range: 6.5–12.2).

Both sites of the SUB ports -were prepared aseptically and a urine sample was taken under sterile conditions by puncture of the ports chambers with a Huber point needle (Norfolk Vet Products, Skokie, USA). Urine analysis was performed and revealed a specific gravity of 1.012, pH 5.1, hematuria, pyuria, and no visible bacteria on the sediment examination. The rest of the sample was submitted for routine culture at 37 °C on Trypticase Soy Agar II with 5% Sheep Blood (BD, Heidelberg, Germany) to detect bacteria able to cause cystitis. No bacterial colonies had grown after 24 h of incubation, however confluent growth was observed after 96 h and the colonies were identified as *F. tularensis* by MALDI-TOF (Bruker, Bremen Germany) using an in-house database. Since this was a highly unusual finding, the treating veterinarian was asked to submit a second sample, which was subjected to culture and Real-time PCR [8], both of which were again positive for *F. tularensis*.

The cat was treated with doxycycline 50 mg once daily for 3 weeks and urine samples for PCR were again collected by puncture of the SUB ports in June, August and September 2019 and again in June 2020. Starting from June 2019, culture results were negative, however PCR remained positive until September 2019 albeit with lower *C*_t_-values (Table 1). Treatment with doxycycline had been restarted mid August 2019 for a total of 2 months due to persistent positive PCR and concern for possible zoonotic risk for the owners. A serum sample was obtained from the cat on 20-Jun-19 (6 weeks after initial urine sampling) and sent to the FLI Friedrich-Löffler-Institut (Jena, Germany) for a microagglutination Test, which resulted in a low positive titer (Table 1).

**Table 1:** **Diagnosis of tularemia based on urine samples and serum from a domestic cat.**

Sampling date	Culture	qPCR (*C*_t_ value)	Serology
May 9, 2019	Positive	ND	ND
May 14, 2019	Positive	Positive (28)	ND
June 20, 2019	Negative	Positive (28)	1:20
August 8, 2019	Negative	Positive (38)	ND
September 11, 2019	Negative	Positive (36)	ND
June 2, 2020	Negative	Negative (> 40)	ND

To investigate a possible epidemiological relationship with wildlife, the cat isolate and hare isolates derived from cases necropsied in 2019 (Table 2) were subjected to whole genome sequencing and SNP analysis. Illumina 150 bp paired-read sequencing was performed by Eurofins Genomics Germany GmbH (Ebersberg, Germany). On top of the Illumina sequencing PacBio sequencing was performed by the Lausanne Genomics Technologies Facility (GTF) (Lausanne, Switzerland) aiming to make the analyses more accurate. Ambiguous sites were resolved by Sanger sequencing (Microsynth AG, Balgach Switzerland) (primers specified in Additional file 1). The sequence data was submitted to the Sequence Read Archive: PRJNA645814.

**Table 2:** **Swiss Francisella tularensis subsp. holarctica isolates included in the analyses.**

Sampling date	Isolate ID	Specimen	Host	CanSNP clade
March 20, 2019	19OD0470	Liver	Hare	B.45
April 4, 2019	19OD0551	Liver	Hare	B.92
April 11, 2019	19OD0587	Liver	Hare	B.47
April 16, 2019	19OD0665	Liver	Hare	B.61
April 17, 2019	19OD0695	Liver	Hare	B.61
April 18, 2019	19OD0700	Liver	Hare	B.47
April 26, 2019	19OD0758	Spleen	Hare	B.45
May 7, 2019	19OD0847	Liver	Hare	B.61
May 9, 2019	*19KM1151*	*Urine*	*Cat*	*B.53*
May 14, 2019	*19KM1164*	*Urine*	*Cat*	*B.53*
May 14, 2019	19OD0886	Liver	Hare	B.45
May 15, 2019	19OD0902	Liver	Hare	B.45
May 29, 2019	*19OD0988*	*Liver*	*Hare*	*B.53*
June 13, 2019	19OD1266	Liver	Hare	B.46

The cat isolates as well as the hare isolate of the same CanSNP clade are marked in italic.

**Figure 1:**
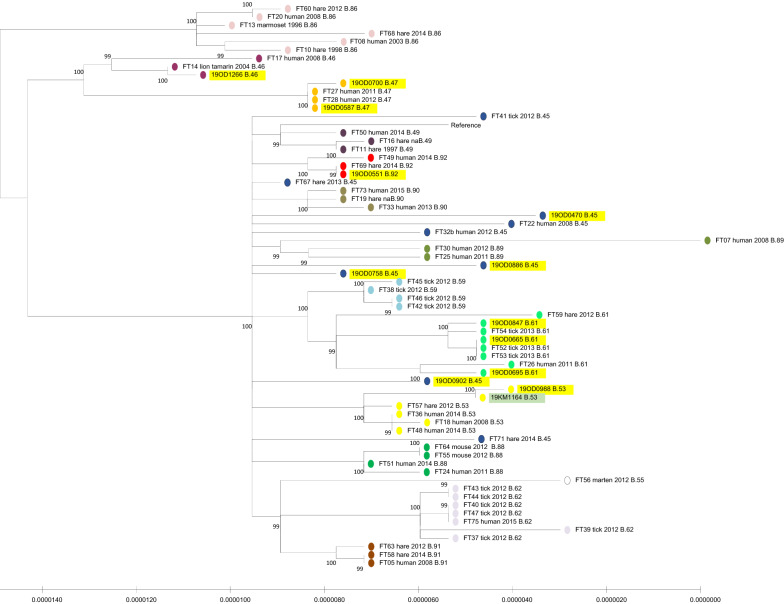
**Maximum likelihood tree estimated using PhyML 3.3.20180214.** Hare strains from the current study are marked in yellow and the cat strain is shown in green. Strains from Wittwer et al. [7] are included for comparison. CanSNP clade B.33 was used as an outgroup (not shown). Only the B.11 cluster is shown, subclades are marked with different colors and indicated for each strain.

**Figure 2:**
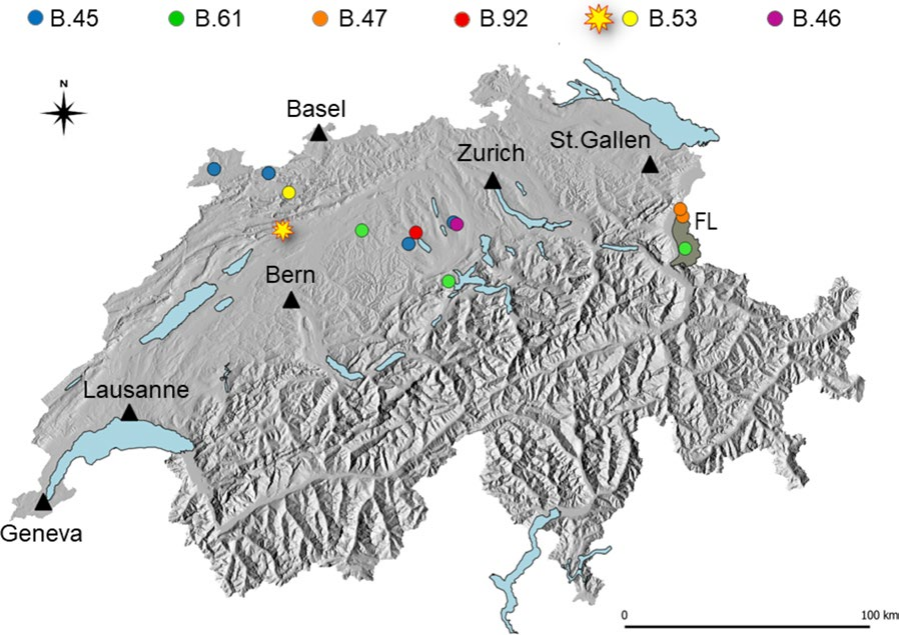
**Map of Switzerland depicting the geographical origin of the investigated animals (hares as circles, cat as a star), with landscape relief (shades of grey), main lakes (pale blue), main cities (black triangles) and the neighboring Principality of Liechtenstein (FL, homogenous grey area).** Different colors attributed to the animals correspond to the identified CanSNP clusters.

Hybrid assemblies with Illumina and PacBio reads were generated using unicycler 0.4.4 [9]. For SNP analyses the snippy 4.4.5 pipline [10] was applied using *F. tularensis* subsp. *holarctica* FTNF002-00 (NC_009749.1) as reference. Furthermore, assemblies of strains from Swiss wildlife, humans and ticks described earlier [7] were downloaded from Genbank and included for comparison. In order to generate phylogenetic trees, at first the best evolutionary model was determined with the program ModelTest-NG 0.1.3 [11], which was followed by tree creation with PhyML 3.3.20180214 [12]. In order to classify isolates in the canonical SNP framework, the program CanSNper 1.0.8 [13] was used. The cat strain was shown to belong to the CanSNP B.53 clade, which is part of the B.11 cluster, the most prevalent in Switzerland [7]. It was very closely related to 19OD0988 isolated in the same month from a brown hare in a neighboring geographical region (Figures 1 and 2).

## Discussion

Tularemia is a life-threatening zoonotic disease if untreated, which has been increasingly reported in humans in several European countries [3]. In Switzerland, the incidence of tularemia in humans has risen from 0.18/100 000 in 2010 to 1.72 in 2019 [14]. A large outbreak was observed in Sweden in 2019 [15] and in Germany tularemia is also considered a re-emerging disease [16]. A higher frequency of tularemia cases can be related to more sensitive diagnostic procedures, increasing surveillance or spread of the pathogen. Over the last years, *Francisella*, has also been increasingly isolated from small rodents and hares in Switzerland [17] even though the trend is not as clear as for humans. A higher prevalence in wildlife might also favor spillover events to domestic animals. Since small rodents are part of the diet of domestic cats, oral infection is possible. This is a plausible hypothesis given the fact that the here described cat was reported to be an active hunter. Another possibility would be transmission via ticks, which have been shown to harbor the same isolates as hares and humans [7].

The cat isolate showed a close phylogenetic relationship to a contemporary isolate from a hare that was found in geographical vicinity. This fact supports an epidemiological link between these cases, either directly (contact with small rodents, arthropod vectors) or indirectly (environmental contamination).

Even though, to the best of our knowledge, feline tularemia has not been previously reported in Europe, there are reports of human tularemia following cat bites [18, 19]. This might indicate a transmission via saliva or an undiagnosed infection of the cat. The case described here is also unique in its unexpected clinical presentation as subclinical bacteriuria. However, urine of cats is not routinely analyzed for *Francisella* and thus previous cases may have been missed due to the fastidious nature and slow growth of the organisms. It remains unclear as to whether infection contributed to the weight loss observed in this cat in the absence of further disease signs. Tularemia in cats caused by subspecies *tularensis* has been described to manifest as a systemic illness with fever, apathy, lymphadenopathy, hepato-splenomegaly or as oral ulcers after ingestion of infected rodents [20, 21]. While tularemia can cause lesions in the renal pelvis as part of a systemic infection in hares [22], it is not known to occur solely as a urinary tract infection or colonization.

The cat quickly became culture negative after treatment with doxycycline even though increased minimal inhibitory concentrations and treatment failures have been described for *Francisella* [23]. The positive PCR results over a long time may be due to persistence of dead organisms or DNA in the SUB system.

In 2014, the first case of tularemia caused by subspecies *holarctica* in a European gun dog was reported from Norway [24]. The dog and his owner had been exposed to an infected mountain hare during hunting and subsequently both dog and owner developed clinical tularemia [24]. A 2018 study in Austrian hunting dogs found a seroprevalence of 6% with most dogs showing no signs of tularemia [25]. In the present cat case, fortunately, none of the exposed humans including owner and veterinary staff were clinically affected, however, no testing was performed. Nevertheless, shedding of *Francisella* in urine poses a considerable risk for owners, veterinary staff and other people through the contamination of the environment. Most veterinary diagnostic laboratories are not prepared to isolate and identify *F. tularensis* from urine*.* Due to its classification as a select agent, it is not present in the standard MALDI-TOF databases used by many veterinary laboratories. The inclusion of *F. tularensis* in the standard MALDI-TOF database would foster detection of infections in unusual hosts and overall detection of the pathogen, resulting in a better picture of its geographical distribution and host range.

In conclusion, this is the first report of tularemia in a domestic cat in Europe and of a presentation as subclinical bacteriuria. Future studies in regions with increased tularemia should include domestic carnivores as potential source of infection for humans. Furthermore, urine should be considered a possible source of *F. tularensis*, even when originating from asymptomatic animals.

## Supplementary information

**Additional file 1. List of primers used for Sanger sequencing.**

## Data Availability

The datasets generated and analyzed during the current study are available in the Genbank repository under Bioproject accession number PRJNA645814.

## Abbreviations

IRIS: International Renal Interest Society
MALDI-TOF: matrix-assisted laser desorption/ionization time of flight
SUB: subcutaneous ureteral bypass
